# Association between Prenatal One-Hour Glucose Challenge Test Values and Delivery Mode in Nondiabetic, Pregnant Black Women

**DOI:** 10.1155/2015/835613

**Published:** 2015-05-25

**Authors:** Jerel M. Ezell, Rosalind M. Peters, Jessica E. Shill, Andrea E. Cassidy-Bushrow

**Affiliations:** ^1^Department of Public Health Sciences, Henry Ford Hospital, Detroit, MI 48202, USA; ^2^College of Nursing, Wayne State University, Detroit, MI 48202, USA; ^3^Department of Endocrinology, Diabetes, and Bone and Mineral Disorders, Henry Ford Health System, Detroit, MI 48202, USA

## Abstract

*Objective*. We examined the association between 1-hour glucose challenge test (GCT) values and risk of caesarean section. *Study Design*. A prospective cohort study recruited 203 pregnant Black women to participate. At ~28 weeks of gestation, participants underwent a routine 1-hour 50 g GCT to screen for gestational diabetes mellitus. Logistic regression was used to examine the association between 1-hour GCT value and delivery mode. *Results*. Of the 158 participants included, 53 (33.5%) delivered via C-section; the majority (*n* = 29; 54.7%) were nulliparous. Mean 1-hour GCT values were slightly, but not significantly, higher among women delivering via C-section; versus vaginally (107.8 ± 20.7 versus 102.4 ± 21.5 mg/dL, resp.; *P* = 0.13). After stratifying by parity and adjusting for maternal age, previous C-section, and prepregnancy body mass index, 1-hour GCT value was significantly associated with increased risk of C-section among parous women (OR per 1 mg/dL increase in GCT value = 1.05; 95% CI OR: 1.00, 1.05; *P* = 0.045). *Conclusion*. Even slightly elevated 1-hour 50 g GCT values may be associated with delivery mode among parous Black women.

## 1. Introduction

Over the last two decades, the volume of planned and unplanned caesarean section (C-section) deliveries performed globally has risen steeply [1, 2]. Although these rates have begun to plateau in recent years [3], additional research is needed to better contextualize factors associated with C-sections and further drive reductions in the practice [4, 5]. This is especially important as data consistently illustrate that C-sections are associated with a heightened risk of short- and long-term morbidity and mortality in the mother. C-section is also associated with obesity, Type 1 diabetes, and allergic disease in offspring [6–9].

Maternal and fetal factors associated with C-section include older maternal age, greater parity, prior C-section delivery, increased maternal or fetal weight, and breech presentation [10–12]. Racial dimensions also exist: population-based evidence consistently demonstrates that rates of C-sections are disproportionately higher among women in racial/ethnic minority populations, in comparison to White women [13–15]. “Nonclinical” drivers (e.g., personal motivators) of C-sections, including mothers' worries of logistical inconvenience or fears of enduring physical harm or difficulty during or after vaginal delivery, also undergird the rise in the practice [16, 17]. Physician biases may be an additional contributor to the increase in C-sections performed; in a recent survey, nearly 30% of OB/GYNs indicated that they had been increasing the number of C-sections performed over fears of malpractice suits [18].

Several previous studies have shown women with elevated or high-normal values from the 1-hour glucose challenge test (GCT) and oral glucose tolerance test (OGTT), used to screen for gestational diabetes mellitus (GDM), may have an elevated risk of poor perinatal health outcomes, including C-section [19–21], while other studies have been more equivocal [22, 23]. However, to our knowledge, no prior study has examined potential relationships between these factors in a large sample of Black women, a group at elevated risk of C-section in comparison to other racial/ethnic groups [24, 25]. For our primary analysis, we examined the relationship between continuous 1-hour 50 g GCT values and risk of C-section, in a population of nondiabetic, Black women receiving prenatal care from a large, integrated healthcare system in metropolitan Detroit, Michigan, USA. As parity is associated with subsequent delivery mode and a previous study suggested the association between 3-hour OGTT value and delivery mode was modified by parity [21], we also examined if associations between 1-hour 50 g GCT delivery mode varied by parity.

## 2. Methods

### 2.1. Study Protocol

The study population and protocols included in the analysis are described in detail elsewhere [26–28]. Briefly, pregnant Black women between ages 18 and 44, receiving prenatal care from obstetrics clinics in the Henry Ford Health System (HFHS) in metropolitan Detroit, Michigan, USA, between February 2009 and June 2010, were recruited to participate in the study. All study procedures were approved by the appropriate Institutional Review Boards at HFHS and Wayne State University, and written informed consent was obtained from all study participants.

In accordance with the American College of Obstetricians and Gynecologists (ACOG) guidelines [29], as part of routine prenatal care, women were screened for GDM at approximately 28 weeks of gestation using the 1-hour 50 g GCT. Women classified as screening “positive” were then tested for GDM with the 3-hour 100 g OGTT. The lower bound for screening positive depends on the individual medical provider making the determination; at HFHS, the criteria to classify women as screening positive varied slightly (cutoffs of GCT ≥ 130 mg/dL, ≥135 mg/dL, or ≥140 mg/dL are used by different clinicians) [21]. For purposes of analysis, our primary analysis was done using continuous GCT levels; when examining based on categorical considerations, an abnormal GCT screen was defined using the mid-point of the value used at HFHS of ≥135 mg/dL.

The HFHS corporate data store system was queried, using a participant's unique medical record number, to obtain all 1-hour 50 g GCT and 3-hour 100 g OGTT dates and values collected over the course of the pregnancy. Electronic medical record abstraction was performed to obtain delivery and birth information, including data on the following variables: (1) delivery mode (e.g., vaginal versus (planned or unplanned) C-section); (2) labor induction/augmentation; (3) gestational age (GA) at delivery; (4) infant gender; and (5) infant birth weight. Low birth weight (LBW) was defined as a birth weight of <2500 g. Macrosomia was defined as a birth weight of ≥4000 g [30, 31]. Birth weight *Z*-scores were calculated using the US population as a reference [32]. Preterm birth (PTB) was defined as a birth occurring at <37 weeks of GA at delivery.

Of the original sample of 203 women, 19 (9.4%) had either a clinician-documented GDM diagnosis in the current pregnancy (*n* = 12) or 3-hour 100 g OGTT values consistent with GDM (*n* = 7) and were excluded from this analysis. Additionally, 26 women were excluded from analysis if they met any of the following criteria: (1) they had preexisting Type 2 diabetes (*n* = 5); (2) they were never screened for GDM (*n* = 5); (3) they had an incomplete 1-hour GCT (*n* = 2); (4) they had an abnormally high 1-hour GCT result never followed up for diagnostics, due to presentation for labor (*n* = 2); (5) they met the Leykin and Pellis (2009) definition for “super-super” morbid obesity (i.e., maternal prepregnancy BMI > 60 kg/m^2^) (*n* = 3) [33]; (6) they delivered twins (*n* = 2); or (7) there was missing delivery information due to delivery occurring at an outside facility (*n* = 7). The final analytic sample size was *N* = 158.

### 2.2. Statistical Analysis

Participant characteristics were compared by delivery mode (vaginal delivery versus C-section) using Chi-square or Fisher's exact test for discrete covariates and Student's *t*-test for continuous covariates. Pearson correlation coefficients were used to examine the relationship of continuous 1-hour GCT values with continuous participant characteristics. Student's *t*-test was used to examine if there were mean differences in continuous 1-hour GCT values across discrete covariates.

Logistic regression models were fit to examine the association of continuously distributed 1-hour GCT values and delivery mode (vaginal versus C-section). Models were fit unadjusted and then adjusted for potential confounding variables, specifically, maternal age, previous C-section, and maternal prepregnancy BMI, which were identified in the literature as variables associated with delivery mode and/or 1-hour GCT value [21, 25]. We then refit our models stratified by parity status (nulliparous compared to parous) [34].

We also explored the association of categorical 1-hour GCT value with delivery mode, stratified by parity. Additionally, we examined if the association of 1-hour GCT values with delivery mode varied by type of C-section (planned versus unplanned) compared to vaginal delivery.

## 3. Results

### 3.1. Study Population

Participant characteristics are provided by delivery mode in Table 1. Briefly, the mean age of participants at the time of the study visit was 25.9 ± 5.9 years. In total, 76 (48.1%) participants were nulliparous and 2 women reported prior GDM.

More women delivered vaginally (*n* = 105, 66.5%) than by C-section (*n* = 53; 33.5%). In general, characteristics between women who delivered vaginally and those who delivered via C-section were similar (Table 1). However, women who delivered vaginally had a significantly lower prepregnancy BMI compared to those who delivered via C-section (27.2 ± 6.7 kg/m^2^ versus 31.4 ± 7.3 kg/m^2^, resp.; *P* < 0.001). Also, statistically significantly more women delivering vaginally had their labor induced/augmented (*n* = 78; 76.5%) as compared to women delivering via C-section who had labor induced/augmented (*n* = 28; 52.8%) (*P* = 0.003).

**Figure 1:**
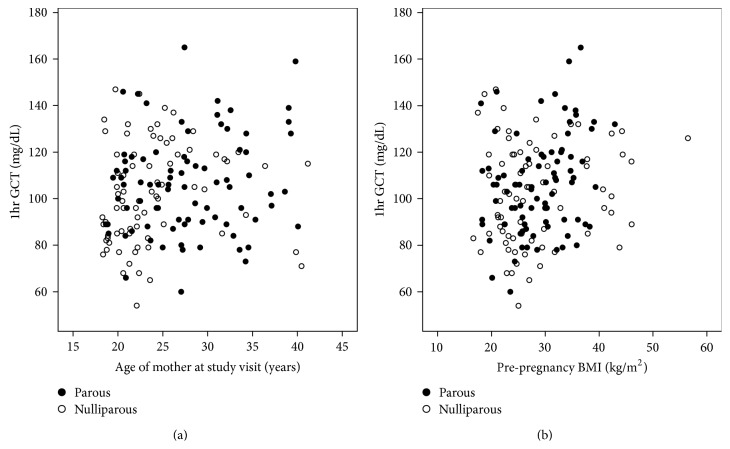
(a) Correlation of 1-hour glucose challenge test (1 hr GCT) value with maternal age at study visit. (b) Correlation of 1-hour glucose challenge test (1 hr GCT) value with maternal prepregnancy BMI.

The overall mean 1-hour GCT value was 104.2 ± 21.3 mg/d. Continuous 1-hour GCT values were significantly and positively correlated with the age of the mother at the study visit (*r* = 0.20; *P* = 0.012) (Figure 1(a)) and with maternal prepregnancy BMI (*r* = 0.18; *P* = 0.018) (Figure 1(b)). Mean continuous 1-hour GCT values were also slightly, but not statistically significantly, higher among parous compared to nulliparous women (105.9 ± 21.1 mg/dL versus 101.9 ± 21.3 mg/dL, resp.; *P* = 0.227). Stratified by parity, continuous 1-hour GCT values were significantly and positively correlated with prepregnancy BMI in parous women (*r* = 0.28; *P* = 0.007), but not in nulliparous women (*r* = 0.13; *P* = 0.261) (Figure 1(b)).

Among nonobese women, the mean continuous 1-hour GCT value was 100.0 ± 20.7 mg/dL, compared to 111.2 ± 20.4 mg/dL among obese women. This difference was statistically significant (*P* = 0.001). Prepregnancy obesity was more common in parous (*n* = 37; 45.1%) compared to nulliparous women (*n* = 22; 28.69%) (*P* = 0.036). Among parous women, continuous 1-hour GCT was statistically significantly higher in women with prepregnancy obesity (112.8 mg/dL) compared to women without prepregnancy obesity (100.7 mg/dL) (*P* = 0.010). In contrast, among nulliparous women, there was not a statistically significant difference in mean continuous 1-hour GCT by prepregnancy obesity status (*P* = 0.130).

### 3.2. Association between Continuous 1-Hour GCT Values and C-Section Delivery

Overall, and in nulliparous women, there was no evidence of an association between 1-hour GCT values and delivery mode (Table 2). In contrast, in parous women, continuous 1-hour GCT values were significantly associated with delivery mode. In the unadjusted model, for every 1 mg/dL increase in 1-hour GCT value, the odds of C-section delivery increased by 1.03 (*P* = 0.034) (Table 2). Results were similar after adjusting for maternal age, previous C-section, and maternal prepregnancy BMI (Table 2).

A total of 13 (8.2%) women had an elevated 1-hour GCT (≥135 mg/dL); 10 of these 13 women (76.9%) completed the 3-hour OGTT, and all 10 passed the 3-hour OGTT test. In the nulliparous group, a total of 3 out of the 47 (6.4%) women who delivered vaginally had an elevated GCT compared to 1 out of the 29 (3.6%) women who delivered by C-section (Fisher's exact test *P* = 0.506). Among parous women, a total of 5 out of the 58 (8.6%) women who delivered vaginally had an elevated GCT, compared to 4 out of 24 (16.7%) women who delivered via C-section having an elevated GCT (Fisher's exact test *P* = 0.244). In contrast to women with 1-hour GCT values <135 mg/dL, parous women with an elevated 1-hour GCT were at 5.1 times higher odds of having a C-section (95% CI 0.7, 37.4; *P* = 0.113), after adjusting for maternal age, maternal prepregnancy BMI, and prior C-section. There was no association between elevated GCT and delivery mode in the nulliparous women.

Mean 1-hour GCT values were higher among women with a planned C-section (114.6 ± 23.3 mg/dL) compared to women with a vaginal delivery (102.4 ± 21.5 mg/dL) or unplanned C-section (104.9 ± 19.1 mg/dL). In multivariable models, there was no evidence of an association between unplanned C-section and 1-hour GCT values (relative to vaginal delivery). Conversely, for every 1 mg/dL increase in 1-hour GCT value, the unadjusted odds of having a planned C-section versus vaginal delivery increased by 1.03 (95% CI: 1.00, 1.05; *P* = 0.036). Adjusting for maternal age, previous C-section, and maternal prepregnancy BMI, the association between 1-hour GCT value and risk of planned C-section compared to vaginal delivery was borderline statistically significant (OR = 1.06; 95% CI: 1.0, 1.12; *P* = 0.051) (models not shown).

Finally, two infants presented in the breech position; we conducted a sensitivity analysis excluding these two deliveries and all model inferences were similar.

## 4. Discussion

In this sample of nondiabetic, pregnant Black women, continuous 1-hour GCT values were associated with delivery mode only among parous women. One-hour GCT values were significantly higher among women with a planned C-section, in comparison to those with an unplanned C-section or vaginal delivery. To our knowledge, no published studies exist which have explored potential associations between 1-hour GCT values, maternal prepregnancy BMI, parity, and delivery mode, specifically in a Black, nondiabetic cohort.

Several other studies have found relationships between increasing glucose values and risk of C-section [21, 35–37]. However, these studies have often failed to include racially diverse samples or examine racial differences in outcomes. One prospective analysis of predominantly Hispanic pregnant women in San Antonio, Texas, determined that there was a positive relationship between GCT value and C-section, in both women who were obese (maternal prepregnancy BMI ≥ 27.3 kg/m^2^) and women who were nonobese (maternal prepregnancy BMI < 27.3 kg/m^2^) [35]. Interestingly, in that study, the increased C-section rate was present only among nonobese women with above-normal GCT values, while, among obese women, an increase in C-section rate was present only among those with normal GCT values [35]. In a large retrospective cohort study, 9% of women screened positive on the 1-hour GCT but were later shown not to have GDM based on 3-hour GCT [19]. These screen-positive women were at a higher risk of C-section [19]. In addition, these women also tended to possess a higher BMI and have greater parity [19], further highlighting the degree to which maternal weight and other maternal health indicators may influence delivery and birth outcomes.

We observed that maternal prepregnancy BMI and continuous 1-hour GCT values were significantly and positively correlated in parous women, but not in nulliparous women. Evidence suggests that maternal prepregnancy BMI is higher among parous women as compared to nulliparous women [38] and that pregnancy-acquired weight gain, particularly in Black women, may persist over time and become permanent [39]. Though limited, research also indicates that weight gain acquired from prior pregnancies, and prepregnancy weight gain, may predict impaired glucose tolerance or GDM in subsequent pregnancies [40–42].

Data suggests that women who are informed of having, or being at elevated risk of, GDM may better control weight gain during pregnancy, in ways comparable to overweight/obese patients who are cautioned against excessive weight gain [43–45]. Recommendations developed in 2010 by the International Association of Diabetes and Pregnancy Study Groups (IADPSG) [46], influenced by findings from the Hyperglycemia and Adverse Pregnancy Outcome (HAPO) Study [36], encouraged clinicians to administer the 75 g OGTT between 24 and 28 weeks of gestation, in an effort to better target attendant glucose-related complications. These recommendations, however, have not been endorsed by ACOG [47]. Whether the 75 g GCT would allow for better risk stratification with respect to C-section is not fully known; however, the single test may enable women to more accurately gauge their own personal risk [48]. A large prospective analysis conducted in Spain revealed that usage of GDM diagnosis criteria from the one-step IADPSG resulted in statistically significant reductions in gestational hypertension, prematurity, and C-section and created substantial overall cost savings [48].

It is important that clinicians carefully modulate their recommendations to ensure that women who have an elevated, 1-hour GCT value but a normal follow-up 3-hour OGTT value have a proper understanding of what the initial positive screening means. Data suggest that treating women with an abnormal 1-hour 50 g GCT, but a normal 3-hour 100 g OGTT, may lower the risk of repeat C-section and macrosomic infants and ultimately prove cost-effective [49, 50].

African American women are at a higher risk of C-section delivery than White women [51, 52]. Two recent studies have identified potential factors associated with the racial disparity in this increased risk, including labor induction among nulliparous women, greater maternal age among parous women, and hypertension [51, 52]. In contrast, we found that a combined labor induction/augmentation variable was associated with decreased risk of C-section; this difference may be attributable to including augmentation of labor in our definition. To our knowledge, however, no studies have considered 1-hour GCT results as a potential factor explaining racial disparities in C-section rates. Roth and Henley [13] have suggested that racial disparities in C-section rates may be accounted for by lack of maternal education necessary to advocate for oneself to avoid a medically unnecessary C-section; whether this extends to there being a sufficient level of medical literacy required to understand an abnormal 1 hr GCT result requires further study.

There are some limitations to the analyses undertaken in the current study. First, the sample size was relatively small; although we were able to stratify by parity, we may be subject to findings due to chance, and thus these findings need to be replicated in other studies. Particularly for the analysis of dichotomized 1-hour GCT value, we may have been underpowered to detect true associations. In addition, we did not have access to information on any recommendations that the involved clinician may have made with respect to the initial 1-hour GCT. Clinicians varied in their use of the 1-hour GCT cutoffs to define a screen positive (varied between 130 and 140 mg/dL) which makes definition of a screen positive difficult. Some women who had values within this range but were not deemed screen positive by their provider may have actually had GDM. For our primary analysis, we relied on the continuous 1-hour GCT results, which diminishes the risk of misclassification based on categorical cut-points. However, examining elevated GCT (≥135 mg/dL) with delivery mode, parous women with elevated 1-hour GCT had a nonsignificant, but large, 5.1 times increased risk of C-section; all but one of these parous women with elevated GCT completed (and passed) the 3-hour OGTT. At least in consideration of parous women, this finding potentially illuminates that, even among women who pass the 3-hour OGTT, merely having an* elevated* GCT value may be predictive of subsequent C-section delivery.

In conclusion, our findings contribute new information regarding the relationship of glucose levels, maternal prepregnancy BMI, parity, and delivery mode, in a high-risk population group, Black women. Specifically, results from our analysis suggested that 1-hour 50 g GCT values may be related to delivery mode among parous, nondiabetic Black women, even after adjusting for the potential confounders of maternal age, previous C-section, and maternal prepregnancy BMI.

## Figures and Tables

**Table 1:** Study participant characteristics (*N* = 158), by delivery mode.

Maternal/delivery characteristics	Vaginal delivery (*N* = 105; 66.5%)	C-section delivery (*N* = 53; 33.5%)	*P*
	Mean ± SD or *n* (%)	Mean ± SD or *n* (%)	
Age at study visit (years)	25.9 ± 6.1	26.1 ± 5.6	0.825
Prepregnancy BMI (kg/m^2^)	27.2 ± 6.7	31.4 ± 7.3	^*∗*^ < 0.001
Prepregnancy BMI category			^*∗*^ < 0.001
Underweight	6 (5.7%)	1 (1.9%)	
Normal weight	37 (35.2%)	12 (22.6%)	
Overweight	35 (33.3%)	8 (15.1%)	
Obese	27 (25.7%)	32 (60.4%)	
Education (years)	12.8 ± 1.8	12.9 ± 1.4	0.888
Annual household income ($)^a^	31,676.5 ± 26,213.6	36,770.5 ± 36,069.5	0.351
Marital status			0.189
Married/living as married	21 (20.0%)	16 (30.2%)	
Separated/divorced	3 (2.9%)	0 (0.0%)	
Never married	81 (77.1%)	37 (69.8%)	
Nulliparous	47 (44.8%)	29 (54.7%)	0.237
Smoked during pregnancy	9 (8.6%)	4 (7.5%)	0.545
Previous C-section	3 (2.9%)	15 (28.3%)	^*∗*^ < 0.001
Labor induced/augmented	78 (76.5%)	28 (52.8%)	^*∗*^0.003
Previous GDM	1 (1.0%)	1 (1.9%)	0.560
Continuous mean 1-hour GCT value (mg/dL)	102.4 ± 21.5	107.8 ± 20.7	0.130
Gestational age at GCT (weeks)	25.9 ± 5.0	25.6 ± 6.9	0.802
Preeclampsia	5 (4.8%)	6 (11.3%)	0.126
Unplanned C-section	N/A	37 (69.8%)	N/A
Breech presentation	0 (0.0%)	2 (3.8%)	N/C
Birth weight (g)^d^	3195.7 ± 509.5	3109.5 ± 703.2	0.387
Birth weight *Z*-score^e^	−0.49 ± 0.90	−0.50 ± 0.90	0.925
Low birth weight^d^	7 (6.9%)	7 (13.5%)	0.151
Infant male gender^b^	46 (43.8%)	29 (54.7%)	0.210
Gestational age at delivery (weeks)^e^	39.1 ± 1.8	38.7 ± 3.2	0.254
Preterm birth^c^	6 (5.7%)	6 (11.3%)	0.177

^a^88 (vaginal delivery) and 46 (C-section) with complete information.

^b^102 (vaginal delivery) and 52 (C-section) with complete information.

^c^104 (vaginal delivery) with complete information.

^d^101 (vaginal delivery) and 52 (C-section) with complete information.

^e^101 (vaginal delivery) and 51 (C-section) with complete information.

^*∗*^Statistically significant result (*P* < 0.05).

N/C: not calculable due to empty cells.

**Table 2:** Association between continuous 1-hour GCT values and delivery by C-section, compared to vaginal delivery, in the overall sample, and stratified by parity.

	Overall		Nulliparous		Parous	
	OR (95% CI)	*P *	OR (95% CI)	*P *	OR (95% CI)	*P *
Model 1						
1-hour GCT (mg/dL)	1.01 (1.00, 1.03)	0.131	1.00 (0.98, 1.02)	0.856	1.03 (1.00, 1.05)	^*∗*^0.034
Model 2						
Maternal age (years)	0.96 (0.90, 1.03)	0.259	1.04 (0.95, 1.14)	0.368	0.90 (0.80, 1.02)	0.896
Previous C-section	15.6 (4.0, 60.2)	^*∗*^ < 0.001	N/A	—	66.8 (10.5, 422.7)	^*∗*^ < 0.001
1-hour GCT (mg/dL)	1.01 (1.00, 1.03)	0.171	1.00 (0.98, 1.02)	0.943	1.05 (1.01, 1.09)	^*∗*^0.017
Model 3						
Maternal age (years)	0.94 (0.87, 1.00)	0.084	1.02 (0.93, 1.11)	0.751	0.86 (0.75, 0.99)	^*∗*^0.039
Previous C-section	15.2 (3.8, 61.4)	^*∗*^ < 0.001	N/A	—	72.4 (9.7, 540.0)	^*∗*^ < 0.001
Maternal prepregnancy BMI (kg/m^2^)	1.09 (1.03, 1.15)	^*∗*^0.003	1.07 (1.00, 1.13)	^*∗*^0.047	1.13 (1.00, 1.27)	^*∗*^0.045
1-hour GCT (mg/dL)	1.01 (0.99, 1.03)	0.356	1.00 (0.98, 1.02)	0.884	1.05 (1.00, 1.05)	^*∗*^0.029

^*∗*^Statistically significant result (*P* < 0.05).

## Abbreviations

ACOG: American College of Obstetricians and Gynecologists
BMI: Body mass index
C-section: Caesarean section
GA: Gestational age
GCT: Glucose challenge test
GDM: Gestational diabetes mellitus
HFHS: Henry Ford Health System
IADPSG: International Association of Diabetes and Pregnancy Study Groups
LBW: Low birth weight
OGTT: Oral glucose tolerance test
PTB: Preterm birth.
